# Translation and Validation of the Diabetes Knowledge Questionnaire in Indonesian Patients With Type 2 Diabetes

**DOI:** 10.1177/26350106241287445

**Published:** 2024-10-19

**Authors:** Indriastuti Cahyaningsih, M. Rifqi Rokhman, Nurul Maziyyah, Eva Niamuzisilawati, Katja Taxis, Petra Denig

**Affiliations:** Unit of PharmacoTherapy, Epidemiology, and Economics, University of Groningen, Groningen, the Netherlands; Department of Pharmacist Profession Education, Faculty of Medicine and Health Sciences, Universitas Muhammadiyah Yogyakarta, Yogyakarta, Indonesia; Department of Health Sciences, Unit of Global Health, University of Groningen, University Medical Center Groningen, the Netherlands; Faculty of Pharmacy, Universitas Gadjah Mada, Yogyakarta, Indonesia; Department of Pharmacist Profession Education, Faculty of Medicine and Health Sciences, Universitas Muhammadiyah Yogyakarta, Yogyakarta, Indonesia; Endocrine Metabolic Diabetes Division, Dr Moewardi Hospital, Surakarta, Central Java, Indonesia; Unit of PharmacoTherapy, Epidemiology, and Economics, University of Groningen, Groningen, the Netherlands; Department of Clinical Pharmacy and Pharmacology, University of Groningen, University Medical Center Groningen, Groningen, the Netherlands

## Abstract

**Purpose:**

The purpose of the study was to translate and cross-culturally adapt the 24-item Diabetes Knowledge Questionnaire (DKQ) for Indonesian patients with type 2 diabetes (T2D) and evaluate its psychometric properties.

**Methods:**

Forward-backward translation, adaptation involving 7 experts, and pretesting to develop the Indonesian version of DKQ were conducted. Psychometric analysis was carried out among T2D patients from 40 primary health care centers in Indonesia. Known-group, convergent and discriminant validity, internal consistency and test-retest reliability were assessed. Additionally, a descriptive item analysis was conducted.

**Results:**

In total, 39 patients participated in the pretesting and 304 patients in the validation process and descriptive analysis. Of the 24 items, 2 were adjusted during the adaptation process, and 1 item was deleted because it did not adequately reflect the original item. Known-group validity was demonstrated because patients with younger ages, higher educational levels, and longer diabetes duration had significantly higher DKQ scores. Convergent validity was demonstrated by a significant positive correlation of the DKQ scores with overall treatment satisfaction. The 23-item DKQ Bahasa Indonesia showed satisfactory internal consistency (Cronbach’s α = 0.73; omega total = 0.72) and good test-retest reliability (intraclass correlation coefficient = 0.87 in a sample of 27 patients). No floor and ceiling effects were found in the item analysis.

**Conclusion:**

The study demonstrates adequate validity and reliability of the 23-item DKQ Bahasa Indonesia for assessing diabetes knowledge in Indonesian primary care patients with T2D. This instrument can be used to identify room for improvement and develop diabetes education programs.

Diabetes mellitus is a chronic disease that has become a worldwide concern. The global diabetes prevalence in 2045 is estimated to be 12.2%, rising from 10.5% in 2021, with 90% of cases being type 2 diabetes (T2D).^1^ Similar increases are reported from Indonesia.^2^ In fact, diabetes was the third cause of death in 2019 in Indonesia, and the burden of health care costs are high to treat patients with diabetes and its complications.^3,4^

Patients’ knowledge about diabetes has been found to be associated with better clinical outcomes.^5^ Thus, the assessment of knowledge is an important step when developing diabetes education programs and evaluating their effectiveness. The Diabetes Knowledge Questionnaire (DKQ) is one of the tools to assess knowledge of diabetes developed by Starr Country Diabetes Education Study. Originally, the DKQ consisted of 60 items but has been simplified into a 24-item version and tested for its validity and reliability.^6^ The 24-item version focuses on patients’ knowledge about diabetes and its management, including diet, taking medications, doing physical activities, being aware of symptoms, monitoring glucose levels, and performing routine food/wound care.^6^ The DKQ has been translated into many languages and tested for item clarity, item difficulties, and item discriminations during translation or validation processes.^5,7-9^ The DKQ has “yes,” “no,” and “I do not know” answer options, which are seen as suitable for patients of diverse backgrounds, including patients with low literacy levels. The DKQ is considered a valid instrument to assess the levels of diabetes knowledge in cross-sectional studies or to measure changes of patients’ diabetes knowledge after receiving diabetes education.^6^ A recent systematic review reported that the psychometric properties of the DKQ have been extensively evaluated, making it the recommended tool for assessing diabetes knowledge in patients with diabetes.^10^ The DKQ is an easily administered instrument that can be distributed on paper for self-completion or as an interview.

The DKQ has been translated and tested in a small sample of Indonesian patients in a previous study,^11^ but the translation process and cross-cultural adaptation were not described, limiting the value of this version. Careful translation and cross-cultural adaptation are crucial steps before using an instrument because there are potential differences across cultural and ethnic groups in the interpretation of words and terminology.^12^

The primary purpose of the study was to translate and cross-culturally adapt the 24-item DKQ for Indonesian patients with T2D and evaluate its validity and reliability. The secondary aim was to explore the variation in response of this DKQ Bahasa Indonesia in a large group of patients with T2D in Indonesia.

## Methods

### Study Procedure

**Figure 1.**
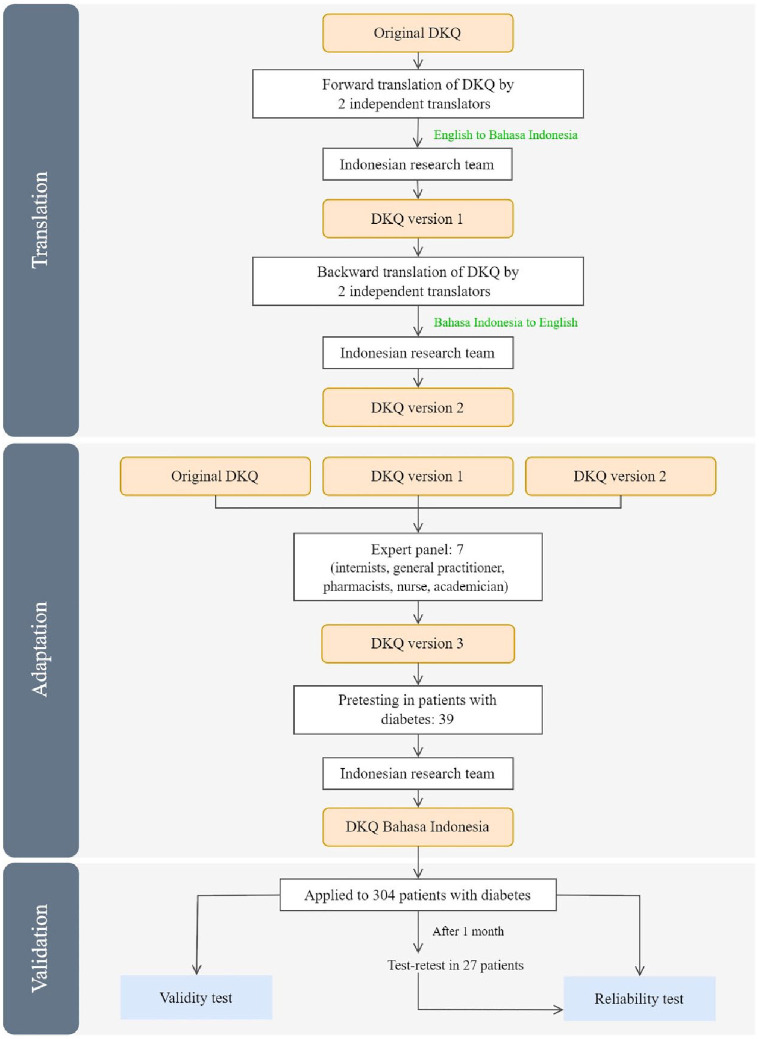
Flowchart of translation and validation process. Abbreviation: DKQ, Diabetes Knowledge Questionnaire.

Based on recommendations for translation and validation of questionnaires in cross-cultural research, the following steps were carried out: translation and cross-cultural adaptation, pretesting, and testing of psychometric properties (Figure 1).^13^ In addition, an item analysis was performed to describe the variation in responses of the translated DKQ among Indonesian patients with T2D.

### Translation and Cross-Cultural Adaptation Process

The original DKQ was translated by 2 independent translators whose mother tongue is Indonesian. One translator had a medical background and was familiar with the terminology of the area covered by the instrument. The second translator had no medical background. The Indonesian team researchers (IC, RR) then compiled and reviewed the translated versions (DKQ version 1). Subsequently, this version was translated back into English by 2 other independent translators who had no medical background. The Indonesian team researchers (IC, RR) again compiled and reviewed the results of these two 2 (DKQ version 2) before it was used in the adaptation process.

Seven experts (2 internists, 1 general practitioner, 2 pharmacists, 1 nurse, and 1 academician having experience in the validation of instruments) assessed the conceptual equivalence (meaning and clarity) between the original DKQ, DKQ version 1, and DKQ version 2. They assessed each item by giving points for equivalence of meaning and clarity such that 1 indicated the item was equivalent or clear and 0 indicated this was not the case. This was done to ascertain that the content of the original questionnaire was maintained. Items that failed to get absolute points of clarity or equivalence were discussed.^9^ The experts were asked to give recommendations for adjusting these items, which were discussed until consensus was reached. Items were then considered to have content validity (DKQ version 3).

Next, clarity and interpretation of the items of DKQ version 3 were tested in face-to-face interviews with patients. During pretesting, respondents were asked whether each item was easy to understand, and they were asked to repeat it using their own words. These interviews were recorded and evaluated by the interviewer and 1 of the researchers (IC). The 2 assessed whether patients sufficiently understood the items by scoring 1 if the explanation of the item was correct and 0 if patient had difficulties in explaining the item. This was used to further adapt items that were not clear or not well understood. This resulted in the final translated version of the DKQ, hereinafter called the “DKQ Bahasa Indonesia.”

### Testing of Psychometric Properties

The validity of the DKQ Bahasa Indonesia was tested by evaluating known-group validity by comparing diabetes knowledge among subgroups of participants based on age, educational level, duration of diabetes, and glycemic status. The hypothesis was that patients with a higher educational level (ie, high school or higher), younger age (ie, less than 55 years old), a longer history of having diabetes (ie, more than 5 years), and controlled glycemic status would have higher scores of diabetes knowledge.^14-16^ Regarding glycemic status, participants were classified as controlled when their A1C levels were <7%, fasting plasma glucose levels were 80 to 130 mg/dL, or oral glucose tolerance tests were <180 mg/dL.^17^

Convergent and discriminant validity were measured by comparing diabetes knowledge with treatment satisfaction as measured by the Diabetes Treatment Satisfaction Questionnaire (DTSQ)^18^ and quality of life as measured by the EuroQol 5-Dimension (EQ-5D) questionnaire.^19^ The DTSQ is a questionnaire widely used in the field of diabetes. It is relatively easy to answer and places a small burden on patients. In addition, it is officially approved by the World Health Organization and the International Diabetes Federation.^20^ Likewise, EQ-5D has wide utility in both clinical and health economic evaluations, where it is useful in demonstrating changes in disease activity and disability.^21^ Additionally, these 2 questionnaires are available in many languages, including Indonesian.

It was hypothesized that diabetes knowledge might have a weak positive correlation with overall treatment satisfaction based on previous findings that patients having received diabetes education had a higher treatment satisfaction.^22^ On the other hand, based on previous studies, no significant correlation between diabetes knowledge and the EQ-5D index score was expected.^23,24^

Internal consistency was tested by calculating Cronbach’s alpha, which is commonly used. However, because it has been recommended to use omega total, internal consistency was also measured using omega total. The omega total value estimates the true reliability by correcting the alpha value because it does not need assumptions, as the Cronbach’s alpha does.^25^ Test-retest reliability was conducted by repeating the measurement at a 1-month interval in a sample of patients, who were selected by random sampling.^26^

In addition, an item analysis was conducted to evaluate the variation in response to the DKQ Bahasa Indonesia by measuring ceiling and floor effects, distribution of DKQ scores, and item difficulty. Ceiling effects occur when many respondents score the highest possible score and cluster around a score of 23 (participants answered all items correctly). Floor effects happen when many respondents score the lowest possible score and cluster around a score of 0 (participants answered all items incorrectly). When the majority of respondents reach either the maximum or the minimum score, the ability of the instrument to capture changes in patient’s diabetes knowledge as a result of an intervention is limited.

The distribution of DKQ scores was presented in frequencies and percentages of each score from the minimum to maximum score. Item difficulty was assessed by calculating the number of correct responses to each item of DKQ divided by the total number of participants.^8^

### Setting and Participants

For pretesting, T2D patients with different educational levels, representing both males and females and different age groups (18 years old or older), were recruited in the study centers. The study was conducted in 10 primary health care centers in 3 districts in Yogyakarta, Indonesia. Patients visiting these centers were recruited by research assistants. The inclusion criteria for pretesting and psychometric testing were T2D patients ages 18 years or older, treated with at least 1 glucose-lowering medication for at least 6 months, able to speak Indonesian, and willing to participate in the study. The exclusion criteria were patients who were unable to communicate effectively.

In the pretesting process, at least 30 participants were aimed to be included.^13^ For testing of the psychometric properties, a minimum of 240 participants was required. This minimal sample is needed to perform the validation of an instrument with 24 items given the recommendation that the minimum number of participants required is 10 times the number of items.^27^

### Statistical Analysis

Descriptive analysis was used to describe the study population. For the known-group validity, the difference in diabetes knowledge between 2 subgroups was assessed using independent *t* test analysis (glycemic status), and analysis of variance tests were used for more than 2 subgroups (age, education, duration of diabetes). Pearson correlation was used to measure the correlation of diabetes knowledge with treatment satisfaction and quality of life in assessing convergent and discriminant validity.

Internal consistency was considered satisfactory when Cronbach’s alpha and omega total have a value ≥0.7.^27^ The test-retest reliability was assessed by calculating the intraclass correlation coefficient (ICC) using a 2-way mixed-effects model with single rater and absolute agreement. This was defined as moderate if the ICC value was between 0.5 and 0.75 and good if the ICC value was >0.75.^28^ ICC reflect the variation in diabetes knowledge between the baseline test and the 1-month retest. Under the assumption that a patient’s knowledge will not change much within this period when no education or intervention is offered, a high ICC value indicates high test-retest reliability.

Ceiling and floor effects and item difficulty and discrimination were presented with descriptive statistics. SPSS version 26.0 was used to perform all statistical analysis. A *P* value lower than .05 was considered a significant difference.

### Ethics Statement

This study was approved by the Health Research Ethics Committee, Faculty of Medicine and Health Sciences, Universitas Muhammadiyah Yogyakarta, Indonesia with No. 153/EC-KEPK FKIK UMY/VII/2022. The authors received grant permission from the American Diabetes Association as a copyright holder to use, adapt, and validate the DKQ instrument with permission request No. KL130722-RR.

## Results

### Cross-Cultural Adaptation

Regarding the content, the experts identified no conceptual equivalence problems (mean score 1 for all items), but 3 items showed issues on clarity (mean scores 0.71, 0.85, and 0.71). The wording of item 23 was recommended as “tight elastic socks” instead of “tight elastic hose or socks,” and item 24 was recommended as “food that is not on the daily menu” instead of “special foods.” Additionally, when checking the final version, the researchers identified 1 conceptual error that was not acknowledged by the experts. This concerned the item 12, “insulin reaction is caused by too much food.” The term “insulin reaction” was incorrectly translated into “insulin starts to work,” whereas this term refers to hypoglycemia or low blood glucose level. Therefore, this item was excluded for further analysis, and a 23-item DKQ Bahasa Indonesia version was used (Table 1).

**Table 1.** Diabetes Knowledge Questionnaire Bahasa Indonesia

No.	Item Pertanyaan	Ya	Tidak	Tidak Tahu
1	Mengkonsumsi terlalu banyak gula dan makanan manis merupakan penyebab diabetes.			
2	Penyebab umum diabetes adalah kerja insulin yang kurang efektif (tidak maksimal) di dalam tubuh.			
3	Diabetes disebabkan oleh kegagalan ginjal menyaring gula agar tidak masuk ke dalam urin (air kencing).			
4	Ginjal menghasilkan insulin.			
5	Jika diabetes tidak diobati, kadar gula dalam darah cenderung meningkat (tidak terkontrol).			
6	Jika saya menderita diabetes, anak saya memiliki kemungkinan lebih tinggi untuk menderita diabetes.			
7	Diabetes dapat disembuhkan.			
8	Gula darah puasa sebesar 210 mg/dL termasuk kadar yang terlalu tinggi.			
9	Cara terbaik untuk memeriksa diabetes adalah dengan tes urin (air kencing).			
10	Olahraga rutin menyebabkan perlunya peningkatan dosis insulin atau obat diabetes lainnya.			
11	Ada dua tipe utama diabetes: Tipe 1 (bergantung insulin) dan Tipe 2 (tidak bergantung insulin).			
12	Untuk mengontrol diabetes, penggunaan obat lebih penting daripada mengatur pola makan dan olahraga.			
13	Diabetes sering menyebabkan gangguan pembuluh/saluran darah.			
14	Pada pasien diabetes, luka lecet dan luka terbuka membutuhkan waktu penyembuhan yang lebih lama.			
15	Pasien diabetes harus lebih berhati-hati saat memotong kuku jari kaki.			
16	Pasien diabetes harus membersihkan luka dengan iodin dan alkohol.			
17	Cara memasak sama pentingnya dengan jenis makanan yang saya makan.			
18	Diabetes dapat merusak ginjal saya.			
19	Diabetes dapat menyebabkan mati rasa pada tangan, jari, dan kaki saya.			
20	Gemetar dan berkeringat merupakan tanda gula darah yang tinggi.			
21	Sering buang air kecil dan merasa haus terus-menerus merupakan tanda gula darah yang rendah.			
22	Kaos kaki yang ketat tidak berdampak buruk untuk pasien diabetes.^a^			
23	Makanan untuk diet pasien diabetes adalah jenis makanan yang berbeda dengan menu sehari-hari.^b^			

a “Hose or socks” was changed into “socks.”

b “Special foods: was changed into “food that is not on the daily menu.”

Thirty-nine patients were included in the pretesting phase (Table 2). All items had mean scores of 0.8 or above, indicating that at least 4 out of 5 patients had no difficulties with explaining the items (Table 3). Some patients had difficulties or were confused about explaining items in their own words. There were, however, no indications or suggestions that a change of wording could increase the clarity of the items for these patients. Based on this pretesting process, no adaptations were made.

**Table 2:** Patient Characteristics in the Pretesting (N = 39)

Characteristic	No. (%)
Gender	
Male	19 (48.7)
Female	20 (51.3)
Age, mean ± SD	62.6 ± 8.6
History of diabetes	
<5 y	18 (46.1)
≥5 y	21 (53.9)
Highest completed educational level	
Elementary school or less	10 (25.6)
Junior or senior high school	25 (64.1)
Bachelor or higher	4 (10.3)
Job status	
Employed	14 (35.9)
Unemployed	25 (64.1)

**Table 3:** Pretesting of Diabetes Knowledge Questionnaire Bahasa Indonesia

Item	Clarity^a^
1	0.8
2	0.8
3	0.8
4	0.8
5	1.0
6	0.9
7	1.0
8	0.9
9	0.9
10	0.8
11	0.8
12	0.9
13	0.8
14	0.9
15	1.0
16	0.9
17	0.8
18	0.9
19	1.0
20	0.9
21	1.0
22	0.8
23	0.9
Mean	0.9

a Mean score of 39 patients (scores 0 or 1).

### Psychometric Properties

For testing the psychometric properties, another 304 patients were included. The majority of patients was female (68.1%), and the mean age was 61.7 ± 8.4. Patients mostly had education at the level of junior or senior high school (53%), were married (97.4%), had unpaid employment (74%), and had a duration of diabetes of less than 5 years (56.3%). The mean DKQ score of patients was 12.9 ± 3.4 (Table 4).

**Table 4:** Patient Characteristics for Testing Psychometric Properties (N = 304).

Characteristic	No. (%)
Age (y), mean ± SD	61.7 ± 8.4
Female	207 (68.1)
Education level	
Elementary or lower	92 (30.3)
High school	161 (53.0)
Bachelor or higher	50 (16.4)
Married	296 (97.4)
Paid employment	79 (26.0)
Body mass index, mean ± SD	25.1 ± 3.9
Duration of diabetes (y)	
<5	171 (56.3)
5-10	71 (23.4)
>10	62 (20.4)
Glycemic status	
Controlled	126 (41.4)
Uncontrolled	178 (58.6)
Diabetes Knowledge Questionnaire, mean ± SD	12.9 ± 3.4

The known-group validity test showed that patients ages below 55 years had better DKQ scores compared to older patients (Table 5). Furthermore, patients having an educational level of high school or higher had better scores of DKQ than those with a lower educational level. Duration of diabetes was associated with DKQ scores. Patients with a diabetes duration of fewer than 5 years had the lowest mean scores. There was no significant difference in DKQ scores between patients with controlled compared to uncontrolled glycemic status. For the convergent and discriminant validity, the Pearson’s correlations showed a significant positive correlation between diabetes knowledge and overall treatment satisfaction (0.154; *P* = .007) but not with the quality of life EQ-5D scores (0.07; *P* = .221), as hypothesized.

**Table 5:** Known-Group Validity of Diabetes Knowledge Questionnaire Bahasa Indonesia

Characteristic	Mean (SD)	*P* Value
Age (y)		
<55	13.6 (3.3)	.011
55-65	13.2 (3.3)	
65-75	12.0 (3.2)	
>75	11.6 (4.0)	
Education		
Elementary or lower	11.1 (3.0)	<.001
High school	13.4 (3.2)	
Bachelor or higher	14.3 (3.6)	
Duration of diabetes (y)		
<5	12.4 (3.3)	.020
5-10	13.1 (3.4)	
>10	13.8 (3.3)	
Blood glucose		
Controlled	12.9 (3.6)	.687
Uncontrolled	12.8 (3.2)	

Internal consistency showed a Cronbach’s alpha value of 0.73 and an omega total of 0.72. Test-retest reliability was conducted in 27 patients, and the ICC value indicated a good reliability (ICC value = 0.87). There was no significant difference between the baseline (mean = 14.5, SD = 2.8) and 1-month retest scores (mean = 14.9, SD = 2.9) of the DKQ Bahasa Indonesia (*P* = .246).

**Figure 2.**
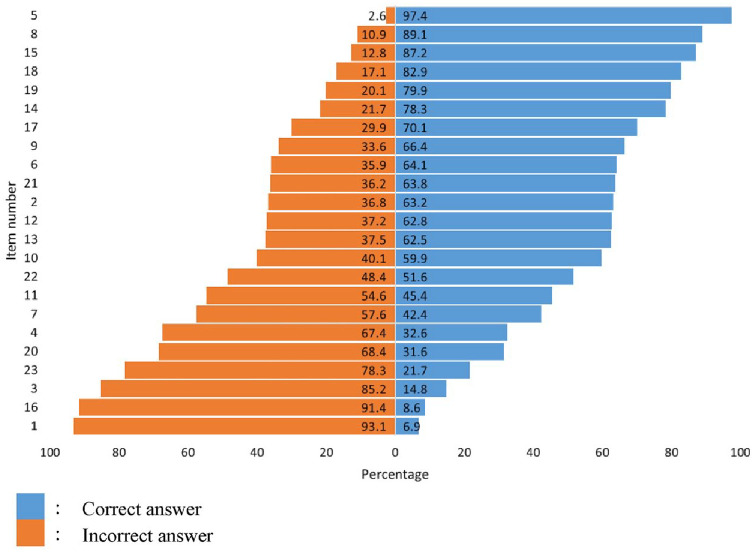
Item difficulty of Diabetes Knowledge Questionnaire Bahasa Indonesia (see Table 7 for Item Descriptions).

None of the patients had all correct or no correct answers for the 23 items, showing no extreme floor or ceiling effects. The mean percentage of correct scores was 55.8 (SD = 26.2), with the minimum and maximum scores of 4 and 21, respectively (Tables 6 and 7). There were 3 items for which fewer than 15% of patients had a correct answer. These were item 1, “Eating too much sugar and other sweet foods is a cause of diabetes”; item 3, “Diabetes is caused by failure of the kidneys to keep sugar out of the urine”; and item 16, “A person with diabetes should cleanse a cut with iodine and alcohol.” Of note, the majority also showed lack of knowledge of the importance of adequate self-care, that is, related to diet and exercise or recognition of hyperglycemia. On the other hand, there were 3 items that more than 85% of patients answered correctly. These were item 5, “In untreated diabetes, the amount of sugar in the blood usually increases”; item 8, “A fasting blood sugar level of 210 is too high”; and item 15. “Diabetics should take extra care when cutting their toenails” (Figure 2; Table 7). Item discrimination, as shown by the item-total correlation, ranged from 0.063 to 0.442 with a mean of 0.26 (Table 7).

**Table 6.** Distribution of Diabetes Knowledge Questionnaire Bahasa Indonesia scores

Diabetes Knowledge Questionnaire Score	Frequency	%
4	2	0.7
5	4	1.3
6	9	3.0
7	11	3.6
8	8	2.6
9	17	5.6
10	20	6.6
11	26	8.6
12	24	7.9
13	48	15.8
14	40	13.2
15	28	9.2
16	29	9.5
17	15	4.9
18	13	4.3
19	7	2.3
20	2	0.7
21	1	0.3
Total	304	100

**Table 7.** Item Analysis of Diabetes Knowledge Questionnaire Bahasa Indonesia

No.	Item	Correct Response No. (%)	Corrected Item-Total Correlation	Cronbach’s Alpha if Item Deleted
1	Eating too much sugar and other sweet foods is a cause of diabetes.	21 (6.9)	0.123	0.740
2	The usual cause of diabetes is lack of effective insulin in the body.	192 (63.2)	0.334	0.728
3	Diabetes is caused by failure of the kidneys to keep sugar out of the urine.	45 (14.8)	0.399	0.722
4	Kidneys produce insulin.	99 (32.6)	0.428	0.719
5	In untreated diabetes, the amount of sugar in the blood usually increases.	296 (97.4)	0.081	0.741
6	If I am diabetic, my children have a higher chance of being diabetic	195 (64.1)	0.136	0.742
7	Diabetes can be cured.	129 (42.4)	0.275	0.732
8	A fasting blood sugar level of 210 is too high.	271 (89.1)	0.143	0.739
9	The best way to check my diabetes is by testing my urine.	202 (66.4)	0.318	0.729
10	Regular exercise will increase the need for insulin or other diabetic medication.	182 (59.9)	0.442	0.717
11	There are two main types of diabetes: Type 1 and Type 2	138 (45.4)	0.359	0.725
12	Medication is more important than diet and exercise to control my diabetes.	191 (62.8)	0.232	0.735
13	Diabetes often causes poor circulation.	190 (62.5)	0.412	0.722
14	Cuts and abrasions on diabetics heal more slowly.	238 (78.3)	0.141	0.740
15	Diabetics should take extra care when cutting their toenails.	265 (87.2)	0.063	0.742
16	A person with diabetes should cleanse a cut with iodine and alcohol.	26 (8.6)	0.179	0.738
17	The way I prepare my food is as important as the foods I eat.	213 (70.1)	0.227	0.735
18	Diabetes can damage my kidneys.	252 (82.9)	0.270	0.733
19	Diabetes can cause loss of feeling in my hands, fingers, and feet.	243 (79.9)	0.128	0.740
20	Shaking and sweating are signs of high blood sugar.	96 (31.6)	0.323	0.728
21	Frequent urination and thirst are signs of low blood sugar	194 (63.8)	0.270	0.733
22	Tight elastic hose or socks are not bad for diabetics.	157 (51.6)	0.413	0.720
23	A diabetic diet consists mostly of special foods.	66 (21.7)	0.306	0.730
	Mean	55.8%	0.26	

## Discussion

The translated and cross-culturally adapted DKQ resulted in a 23-item DKQ Bahasa Indonesia. This instrument showed adequate content validity; known-group, convergent, and discriminant validity; satisfactory internal consistency; and good test-retest reliability to assess diabetes knowledge of Indonesian patients with T2D.

A cross-cultural adaptation process, which was conducted with the involvement of an expert panel and a group of patients, is considered essential to create a translated questionnaire that has a similar concept to the original version and is applicable to the diabetes population based on the culture and characteristics of patients.^12^ In this study, some words were adjusted to better fit the Indonesian culture for 2 items. Unfortunately, both the professional translators and the experts misinterpreted “insulin reaction,” resulting in an item that showed no conceptual equivalence with the original DKQ. A similar incorrect translation was found in other translations of the DKQ, that is, in Urdu and Vietnamese.^5,9^ This indicates that the term “insulin reaction” is not widely known and may also be misinterpreted by patients from various backgrounds when completing the original version of the DKQ. Such differences among multiple translated versions of an instrument have been noted before, for example, between the Scandinavian versions of the Beliefs About Medications Questionnaire, despite the use of thorough procedures, including professional translators and the conduct of a review process.^29^ It was noted that some phrases do not translate well, others may have weaknesses in the original version, and some just do not make sense in certain cultural contexts.^29^ A final harmonization step of intertranslation validity can be helpful to identify such issues.

This study illustrated that diabetes knowledge as measured with the DKQ Bahasa Indonesia can discriminate subgroups based on age, educational level, and history of diabetes. Younger patients, those having a higher educational level, and those with a longer history of diabetes had higher DKQ scores. This is in line with previous findings. Several studies showed that low education was associated with poor diabetes knowledge.^7,9,14-16^ A longer history of the disease can be expected to increase diabetes knowledge because of more exposure to information about the disease and related self-care.^7,14^ On the other hand, this finding that older adults had lower DKQ scores could indicate that the recall of correct information may wane over time.^9,14,15^ The finding is in line with previous studies, where it was suggested that this may be due to deteriorating cognitive function or a generation difference regarding exposure to health education.^14^ This study did not find that diabetes knowledge was associated with better glycemic control. This might be explained by the notion that good glycemic control is influenced by many other factors, including disease severity and medication treatment. One previous study did demonstrate that patients with adequate knowledge of diabetes were more likely to achieve better glycemic control,^16^ but this was not found in 2 other studies.^30,31^

Based on previous studies, we hypothesized that having better diabetes knowledge could be associated with higher diabetes treatment satisfaction^22^ but not likely with a higher quality of life.^23,24^ This was confirmed in this study. However, a recent study did observe a relationship between diabetes knowledge, measured with the Diabetes Knowledge Test, and quality of life.^32^ This may illustrate the complex relationships between diabetes knowledge, self-management, treatment, disease outcomes, and quality of life.

The internal consistency observed in this study with both the Cronbach’s alpha and omega total above 0.7 was considered adequate. For comparison, the DKQ had Cronbach’s alpha of 0.78 in the original version^6^ and Cronbach’s alphas ranging between 0.70 and 0.89 in other languages.^5,7,9^ This study showed good test-retest reliability using a 1-month interval, illustrating that patients gave consistent responses to the DKQ Bahasa Indonesia questionnaire. No large changes in their knowledge in this short time period without receiving feedback on the knowledge score or any specific intervention were expected. Of note, the DKQ has been used in studies with a pre- and posttest design, which showed that it was responsive for measuring large changes after diabetes education.^6,8^

In the Indonesian study population, no floor or ceiling effects were observed for the complete DKQ, that is, no patients having all correct or all incorrect responses. The mean score of the DKQ in this study was in line with previous studies, where it ranged between 12 and 16 out of 24.^5,7,15^ Some items, however, showed high scores and little discrimination. For example, most patients knew that the amount of sugar in the blood increases when diabetes is untreated and also that a fasting blood glucose level of 210 is too high. On the other hand, in line with this study, some previous studies showed that items related to the cause of diabetes, symptoms of high blood sugar, wound and foot care, and diet and physical activities were difficult given that fewer than 60% of patients had a correct response, illustrating room for improvement.^6,15,16^

### Strengths and Limitations

This study has several strengths. First, recommended procedures for translation and including an expert panel to assess content validity using a systematic evaluation of equivalence and clarity were followed. Next, this study also included a patient sample to assess the clarity of all items, using face-to-face interviews, which enabled us to ascertain whether patients only had difficulties in finding their own words or actually had difficulties understanding the item. For the psychometric properties, patients from 10 primary health cares in 3 districts in Indonesia with sufficient numbers of patients were included to support robust results of validity and reliability. Omega total, which has been suggested as providing a more robust estimation of internal consistency than Cronbach’s alpha, was also performed.^25^

A limitation was that the expert panel consisted of only 7 people, who may not all have been sufficiently knowledgeable on the terminology used in the DKQ. This resulted in the deletion of 1 item in the final DKQ Bahasa Indonesia version. The original 24-item DKQ was constructed by retaining all items of the 60-item DKQ that had an item-to-total correlation of 0.25 or more. However, the item about the insulin reaction had a lower item-total correlation but was retained because it had little variability, causing a low item-total correlation. Because the DKQ was not developed to include specific knowledge domains, the deletion of 1 item is not expected to affect the overall knowledge assessment to a large degree. Finally, only 27 patients were included for the test-retest reliability using ICC statistics, limiting the ability to assess poor reliability for individual items.

### Implications for Practice and Research

To provide a 24-item version of the DKQ Bahasa Indonesia, the deleted item should be adapted and tested among Indonesian T2D patients. Given the incorrect translations of this item in other versions of the DKQ, it is recommended to have a harmonization meeting with the developers of the original DKQ. Because the current 23-item version demonstrated adequate validity and reliability, this instrument can be used to evaluate the effectiveness of educational programs intended to improve patients’ knowledge about diabetes.

For clinical practice, the results indicate that there are several areas that Indonesian health care providers should focus on to improve the knowledge of their T2D patients. To support good self-management, it is of particular concern that a considerable number of patients thought their diet needs to consist of mostly special foods and that regular exercise will increase their need for diabetic medication and did not recognize the signs of high blood glucose.

## Conclusion

The study demonstrates adequate content and acceptable validity and reliability of a 23-item DKQ Bahasa Indonesia for assessing diabetes knowledge in patients with T2D in primary health care centers. This instrument can be used to identify room for improvement and develop diabetes education programs. Further work is needed to harmonize translations of the DKQ across the world.
